# Trends and Changing Clinical Profiles in Adolescent Mental Health Inpatient Admissions: A Time-Series and Comparative Study Over the Period 2011–2024

**DOI:** 10.62641/aep.2333

**Published:** 2026-10-15

**Authors:** Reyes Gálvez-Medina, Alicia Martínez-Sebastián, Guillermo Lahera, Álvaro López-Díaz

**Affiliations:** ^1^Mental Health Clinical Management Unit, Regional Public Hospital De La Merced, 41640 Osuna, Spain; ^2^Mental Health Clinical Management Unit, University Hospital Virgen Macarena, 41009 Seville, Spain; ^3^Faculty of Medicine and Health Sciences, University of Alcalá, 28801 Alcalá de Henares, Madrid, Spain; ^4^Research Group on Psychiatric Diseases, Ramón y Cajal Institute for Health Research (IRyCIS), 28034 Madrid, Spain; ^5^Spanish Network for Research in Mental Health, Carlos III Institute (CIBERSAM, ISCIII), 28029 Madrid, Spain; ^6^Translational Psychiatry Research Group (PsyNal), Seville Biomedical Research Centre (IBiS), 41013 Seville, Spain; ^7^Department of Psychiatry, School of Medicine, University of Seville, 41009 Seville, Spain

**Keywords:** adolescent, mental health, inpatient, COVID-19, SARIMA model

## Abstract

**Background:**

This study analysed long-term trends and clinical characteristics of adolescents admitted to an Inpatient Mental Health Unit (IMHU), and examined whether the onset of the coronavirus disease (COVID-19) pandemic modified historical inertia.

**Methods:**

An observational study of IMHU admissions among adolescents aged 11–17 was conducted from 2011 to 2024. Quarterly (Q) admissions were modelled using a pre-pandemic seasonal autoregressive integrated moving average (SARIMA) framework (Q1 2011 to Q1 2020) utilizing all IMHU admission episodes to generate counterfactual forecasts for the period from Q2 2020 to Q4 2024. Excess admissions (observed – expected) were then assessed using a one-sample *t*-test, an exact binomial test and the proportion of quarters falling outside the 95% forecast confidence intervals (CIs). Univariate and multivariable analyses were also performed on unique patients to compare sociodemographic and clinical characteristics in the pre- and post-pandemic periods.

**Results:**

Between 2011 and 2024, a total of 338 admission episodes across 230 unique patients were recorded. Although hospitalisations had shown an upward pre-pandemic trend, post-pandemic admissions episodes exceeded SARIMA forecasts by an average of 2.71 per quarter (95% CI: 0.43–5.00; *p* = 0.023). Observed admissions were above the predicted value in 79% of post-pandemic quarters (*p* = 0.019), while 31.5% of quarters exceeded the upper limit of the 95% forecast CI. Overall, 200 admission episodes were observed compared with 147 expected, representing a relative increase of 36.1%. At the patient level, there was also a significantly higher proportion of adolescent female admissions, a rise in internalising disorders with suicidal behaviour, as well as shorter stays and a greater use of psychopharmacological treatment in the post-pandemic period.

**Conclusions:**

Against a background of increasing adolescent IMHU admissions over time, the pandemic period was associated with a significant excess of admissions episodes beyond expected levels, alongside a shift in their clinical profile.

## Introduction

The effects of the coronavirus disease (COVID-19) pandemic on mental health have been significant, especially among children and adolescents [1, 2]. This age group was particularly vulnerable to the virus control measures because they were in a stage of development marked by heightened neuropsychological sensitivity and greater dependence on their social environments [3, 4]. The extended closure of schools, the suspension of outdoor group activities, and the sudden transition to virtual learning environments reduced opportunities for face-to-face peer interaction and physical activity, which harmed emotional self-regulation, and increased screen time and digital social media use [5, 6]. In many cases, this was exacerbated by the loss of family members to the pandemic, as well as financial difficulties that followed, which exerted a cumulative stress effect on this population [7, 8]. Furthermore, the collapse of the healthcare system and the closure of educational institutions disrupted early detection and referral pathways. This resulted in late presentations to mental health services and greater clinical severity [6, 9]. These factors have precipitated a considerable rise in demand for mental healthcare among underage people since the onset of the pandemic [10]. Thus, visits to paediatric mental health emergency departments have increased by two-thirds, while child and adolescent psychiatric hospitalisations have risen by between 33% and 50%, compared to the expected data prior to the pandemic [11, 12, 13]. More alarmingly, some studies have shown a nearly 75% surge in hospitalisations for self-harm, suicidal thoughts, and attempts in this population [14, 15]. Meanwhile, paediatric hospitalisations due to eating disorders have doubled or even tripled since before the pandemic [14, 16]. These alarming dynamics were closely mirrored in Spain, where the pandemic had a similarly disruptive impact on the mental health of adolescents, leading to an equivalent surge in specialised mental healthcare [17]. The findings therefore indicate a global, sustained, profound and clinically significant impact of the pandemic on child and adolescent mental health, with respect to both quantitative and qualitative changes in the demand for specialised care.

Although the COVID-19 pandemic has revealed that a mental health crisis is occurring among young people, it is important to emphasise that such aggravation didn’t occur ex nihilo. Upward trends in child and adolescent mental health morbidity and in the use of hospital resources had already been observed prior to 2020 [18]. Thus, in most high-income countries, the annual volume of emergency visits for mental health reasons in this age group has increased by approximately 8–10% per year over the past two decades [19, 20, 21]. Simultaneously, the number of adolescents admitted to the Inpatient Mental Health Unit (IMHU) rose by over 50% in the two decades before the pandemic, largely due to an increase in females presenting with self-harm, anxiety, depression, and eating disorders [22, 23, 24]. Analogous pre-pandemic signs of deterioration in adolescent mental health have been identified in Spain, where the rate of psychiatric diagnoses among hospitalised adolescents has increased by 2.5 times since 2000, accounting for nearly 10% of admissions nowadays [13]. This global and regional rise in demand for mental healthcare among children and adolescents may be due to the growing visibility of internalising disorders, which have historically been overlooked compared to externalising disorders [25]. Improved early detection in educational institutions, primary care services and paediatric emergency services may have contributed [26, 27, 28]. Nonetheless, the phenomenon could be attributed to the increased digital exposure experienced by young individuals, such as extensive social media use, cyberbullying, and constant social comparison [29, 30]. Finally, it may also be due to the gradual erosion of the social capital, including declining community activities, weakening neighbourhood support networks, reduced capacity for collective action, and a diminishing sense of belonging [31, 32]. Therefore, the COVID-19 pandemic seems to have acted more as a catalyst than just a trigger, intensifying an existing trend (of increased psychiatric morbidity and demand for care) and modifying the clinical presentation of adolescents accessing mental health services.

In this context, the present study aimed to explore the catalytic role of the pandemic on child and adolescent mental health, focusing on four main objectives: (a) to examine the evolution of hospitalisations of underage adolescents in IMHU from 2011 to 2024 using time-series models, and determine whether the pre-pandemic tendency remained, intensified or mitigated after the irruption of COVID-19; (b) to verify whether the excess hospitalisations were statistically significant and temporally associated with the onset of the pandemic, after controlling for the aforementioned historical inertia and seasonality; (c) to analyse and compare the sociodemographic and clinical profiles of hospitalised adolescents before and after the pandemic; and (d) if changes in the characterisation of this population were observed, to assess whether these clinical features were independently associated with the post-pandemic period. Although previous international studies have addressed these issues, the present study utilised an integrated time-series forecasting framework to differentiate the pandemic-specific phenomena from the pre-existing deterioration in adolescent mental health in Spain. This modelling strategy would allow for a more accurate evaluation of what was truly unique to the pandemic, as opposed to pre-existing regional epidemiological trends.

## Materials and Methods

A retrospective observational study was conducted, including all adolescents aged 11–17 who required admission to the IMHU at Virgen Macarena University Hospital (Seville, Spain) between 2011 and 2024. The hospital serves a population of 575,000 people in southern Spain (105,000 of whom are under the age of 18), distributed in both urban and rural areas. To identify cases of minors admitted to the IMHU, we referred to the Minimum Basic Data Set (MBDS), derived from the register of hospitalised patients during the study period. Age at admission was automatically calculated using the patient’s date of birth and date of hospital admission. Patients not within the 11–17 age range and adolescents admitted to hospital for mental health reasons outside the IMHU (e.g., endocrinology for eating disorders or traumatology, intensive care or internal medicine for complications following suicide attempts) were not part of the predefined sampling frame. Once all adolescents admitted to the IMHU between 2011 and 2024 had been identified, their electronic health records (EHRs) were manually reviewed to extract a set of social, demographic, and clinical variables. The aim was to characterise temporal trends in hospitalisations, the clinical profile of these adolescents, and their health outcomes. To accurately model temporal trends in hospitalisations, a time-series analysis was conducted using all registered IMHU admissions of minors during the study period. In contrast, the sociodemographic and clinical characterisation of minors admitted to IMHU was restricted to unique, individual patients (using data from their first admission within the study period) to avoid the overrepresentation of minors with multiple readmissions. The study was conducted in accordance with the ethical principles of the Declaration of Helsinki and its later amendments, and was approved by the Research Ethics Committee of the Virgen Macarena and Virgen del Rocío University Hospitals (DYNAMICS project; approval no. 1508-N-22). Given the retrospective design and the use of pseudonymised EHR data, informed consent was not required. The study adhered to the Strengthening the Reporting of Observational Studies in Epidemiology (STROBE) guidelines [33].

### Sociodemographic and Clinical Variables

The following data was collected through a retrospective review of the EHR: (1) date of admission (coded by calendar quarter for subsequent temporal modelling); (2) age (years at the time of admission); (3) gender (male or female); (4) ethnicity (European Caucasian vs. other); (5) living arrangements (both parents, single parent, or other arrangements); (6) occupation (employed or in education vs. inactive); (7) relationship status (single vs. in a relationship); (8) first-degree family history of mental disorder (yes/no); (9) trauma history (yes/no); (10) substance use history (yes/no); (11) relevant medical or surgical history (yes/no); (12) history of mental health care (yes/no); (13) primary reason for admission (suicidal behaviour, disruptive behaviour/defiant conduct, or other); (14) treatment approach during hospitalisation (psychotherapeutic, psychopharmacological, or combined); (15) length of stay (days between admission and discharge); (16) primary diagnosis at discharge, according to the International Classification of Diseases 10th Revision (ICD-10); and (17) type of follow-up after discharge from the IMHU (child and adolescent mental health team, community mental health team, or other modalities).

A traumatic history was coded as positive if at least one of the following situations was documented in the EHRs: physical, emotional or sexual abuse, severe negligence, and/or bullying. Substance use in adolescents was defined as harmful or at-risk use of alcohol, tobacco, cannabis, or other illicit drugs before admission. A relevant personal somatic or surgical history was defined as the presence of chronic medical conditions in childhood, such as diabetes, epilepsy, cardiac disease, or chronic respiratory conditions. This also included other medical conditions or surgical interventions which required medical follow-up and significantly impacted daily activities. The primary reason for admission was coded using a hierarchical system with three mutually exclusive categories: (1) suicidal behaviour (observable acts or ideation); (2) severe disruptive behaviour or defiant conduct; and (3) other clinical reasons (such as affective, psychotic, dissociative, or obsessive symptoms, or eating disorder-related behaviours), which justified IMHU admission due to symptom severity when there was no high risk of self- or hetero-aggressive behaviour. The ICD-10 diagnosis at discharge was organised by grouping codes into major diagnostic categories, as in previous epidemiological studies of child and adolescent populations [34].

### Data Analysis

A time-series analysis with quarterly aggregation (Q) was conducted to analyse the temporal evolution (2011–2024) of admissions of minors to the IMHU and estimate the impact of the pandemic period. The second quarter of 2020 (Q2 2020) was defined as the intervention point, coinciding with the onset of the pandemic’s sociosanitary impact. According to this definition, the pre-pandemic period was defined as the timeframe between Q1 2011 and Q1 2020, while the post-pandemic period was defined as the period between Q2 2020 and Q4 2024. To model the evolution of the series and estimate a counterfactual prediction, a seasonal autoregressive integrated moving average (SARIMA) model was employed with the following notation: (p, d, q)(P, D, Q)[S], where (p, d, q) denoted the non-seasonal components of the model; (P, D, Q) corresponded to the seasonal components; and S represented the seasonal period. The model was fitted using data exclusively from the pre-pandemic period, thereby generating a counterfactual projection for subsequent quarters (Q2 2020 to Q4 2024) and estimating the expected values and their 95% confidence intervals (CIs). The most appropriate SARIMA specification was selected by balancing goodness-of-fit and parsimony. The initial candidate models were identified using an automated model selection algorithm, which evaluates stationarity and seasonality to determine the necessary order of differencing and seasonal terms (S = 4). A manual verification of alternative model specifications was subsequently conducted. The candidate models were then compared using the Bayesian Information Criterion (BIC), with the one exhibiting the lowest value being selected as the optimal fit. Model adequacy was assessed using several indicators, including stationary R^2^, root mean square error (RMSE), mean absolute error (MAE) and mean absolute percentage error (MAPE). The independence of model residuals was evaluated using the Ljung–Box Q test. Outlier detection was conducted to minimise distortion in parameter estimation caused by isolated events that were not representative of the underlying structural trend. Any structural outlier that was identified was handled by incorporating it into the model equation as a deterministic dummy variable.

In order to assess the differential impact of the post-pandemic period relative to the expected evolution, several complementary indicators with different levels of statistical stringency were used. The percentage of post-pandemic quarters whose observed values exceeded the upper limit of the 95% forecast CIs was estimated. This was interpreted as a conservative criterion of significant deviation from the counterfactual trend. The average magnitude of excess admissions was assessed using a one-sample *t*-test of the quarterly residuals (defined as observed minus expected admissions). Directional consistency was evaluated using a two-sided exact binomial test, which determined whether the proportion of post-pandemic quarters with observed values above the point forecast was greater than 50%. Furthermore, the cumulative absolute difference between projected and observed admissions during the post-pandemic period was calculated, expressed as a relative percentage increase with respect to the value expected by the model. Finally, an exploratory descriptive sub-period analysis was performed, dividing the post-pandemic period into three clinically and epidemiologically meaningful phases: the acute pandemic and restriction phase (Q2 2020–Q4 2020), the progressive reopening phase (Q1 2021–Q1 2022), and the post-restriction recovery phase (Q2 2022–Q4 2024). Cumulative excess admissions were calculated for each phase by summing quarterly residuals, defined as observed minus expected admissions according to the counterfactual SARIMA model.

The descriptive statistics corresponding to the characteristics of the adolescents admitted to the IMHU were expressed as proportions, means, medians, and standard deviations (SD) or interquartile ranges (IQR), as appropriate. The normality of the quantitative variables was assessed using the Kolmogorov–Smirnov test. Bivariate comparisons were conducted to analyse possible differences in the profiles of adolescents admitted to IMHU before and after the pandemic. Student’s *t*-test, Mann–Whitney U test, Fisher’s exact test (with the Freeman-Halton extension for 2×C tables) and chi-squared test were used to assess statistical differences between groups according to the variable type. Chi-squared and Fisher’s exact association tests were estimated using Monte Carlo simulation with 10,000 replications and a 95% CI. A multivariable logistic regression model was fitted to examine which clinical characteristics were independently associated with the study period (pre- versus post-pandemic). This model was intended to identify features that differed between admissions during the two timeframes, rather than to establish causal or temporal relationships between these variables and the study period. Variables were entered into the model according to clinical plausibility and the results of bivariate comparisons. Where appropriate, variables could be recoded prior to modelling in order to improve parsimony and interpretability. Model fit was assessed using the Hosmer-Lemeshow test, and collinearity between covariates was explored before final model interpretation. Associations were expressed as odds ratios (ORs) with 95% CIs. The threshold for statistical significance was set at *p* < 0.05. Cases with missing data were excluded using pairwise deletion. All statistical analyses were performed using SPSS software (version 29, IBM Corp., Armonk, NY, USA), with the Expert Modeler module used for automated time-series forecasting.

## Results

### Temporal Trends in IMHU Admissions Among Children and Adolescents Before and After the Onset of the Pandemic

In absolute terms, 338 episodes of hospitalisation of minors were registered in the IMHU between 2011 and 2024. From Q1 2011 to Q1 2020, there was a consistent upward trend in the number of quarterly hospitalisations. Therefore, while the mean ranged 2–4 admissions per quarter in the early years of the studied period (2011–2014), in the period immediately preceding the pandemic (2018–2019), figures of 6–10 admissions per quarter were recorded. The time series model selected for the counterfactual estimation was a SARIMA (0,1,1)(1,0,1)[4], which demonstrated an appropriate balance between fit and parsimony, with the lowest BIC of 1.467. This specification indicated that stationarity was achieved through one order of non-seasonal differencing (d = 1) and that seasonality was captured via one seasonal autoregressive parameter (P = 1) and one seasonal moving average parameter (Q = 1). The model fit was satisfactory (stationary R^2^ = 0.552), and the error indicators showed reasonably good values (RMSE = 1.623; MAE = 1.218; MAPE = 45.6%). The MAE corresponded to an average absolute deviation of around 1.2 admissions per quarter, whereas the RMSE remained below two admissions per quarter. Although the MAPE was relatively high, the low admission counts in the early years of the series made percentage-based error metrics such as MAPE sensitive to small-denominator effects. The Ljung–Box Q test (Q[18] = 18.09; df = 15; *p* = 0.258) revealed no significant autocorrelation in the residuals. Only one structural outlier was detected throughout the modelled period, which was successfully neutralised by the intervention of a dummy variable, thereby indicating stability in the fit.

Fig. 1 shows the SARIMA model with the counterfactual projection for the post-pandemic period, alongside the observed admission series for the same period. From Q2 2020 to Q4 2024, the model’s counterfactual projection estimated around 8–10 admissions per quarter. However, it was found that, in six of the 19 post-pandemic quarters (31.5%), the number of admissions exceeded the upper limit of the 95% CIs of the counterfactual prediction. A comparison of this projection with the observed values after the pandemic began revealed a mean excess of 2.71 admissions per quarter during the post-pandemic period (SD = 4.74; 95% CI: 0.43–5.00). A one-sample *t*-test showed that this difference was statistically significant (*t*(18) = 2.49; *p* = 0.023). Furthermore, the two-sided exact binomial test demonstrated that, in 79% of post-pandemic quarters, the observed values were higher than those predicted by the model (*p* = 0.019). Cumulatively, the counterfactual model projected 147 admissions for the post-pandemic period; however, the actual number of recorded admissions was 200, representing a 36.1% relative increase compared to the pre-pandemic historical trend. An exploratory descriptive sub-period analysis of model residuals revealed that, during the acute pandemic and restriction phase (Q2–Q4 2020), observed admissions were lower than expected, with a cumulative excess of –7 admissions. The progressive reopening phase (Q1 2021–Q1 2022) showed a marked increase, with a cumulative excess of 25 admissions. The post-restriction recovery phase (Q2 2022–Q4 2024) showed an overall cumulative excess of 35 admissions. However, this pattern was heterogeneous and appeared to attenuate in 2024, when observed admissions were in line with expected values. Finally, to assess whether these observed temporal trends could have been artificially influenced by a subgroup of frequent adolescent readmitters, the distribution of readmission episodes was examined across the pre- and post-pandemic periods. Overall, 108 of the 338 IMHU admission episodes corresponded to repeated admissions. This proportion was comparable across periods: 31.2% (43 of 138 episodes) in the pre-pandemic period and 32.5% (65 of 200 episodes) in the post-pandemic period (*p* = 0.795), suggesting that the observed excess in the time-series was unlikely to be primarily driven by repeated admissions.

**Fig. 1.**
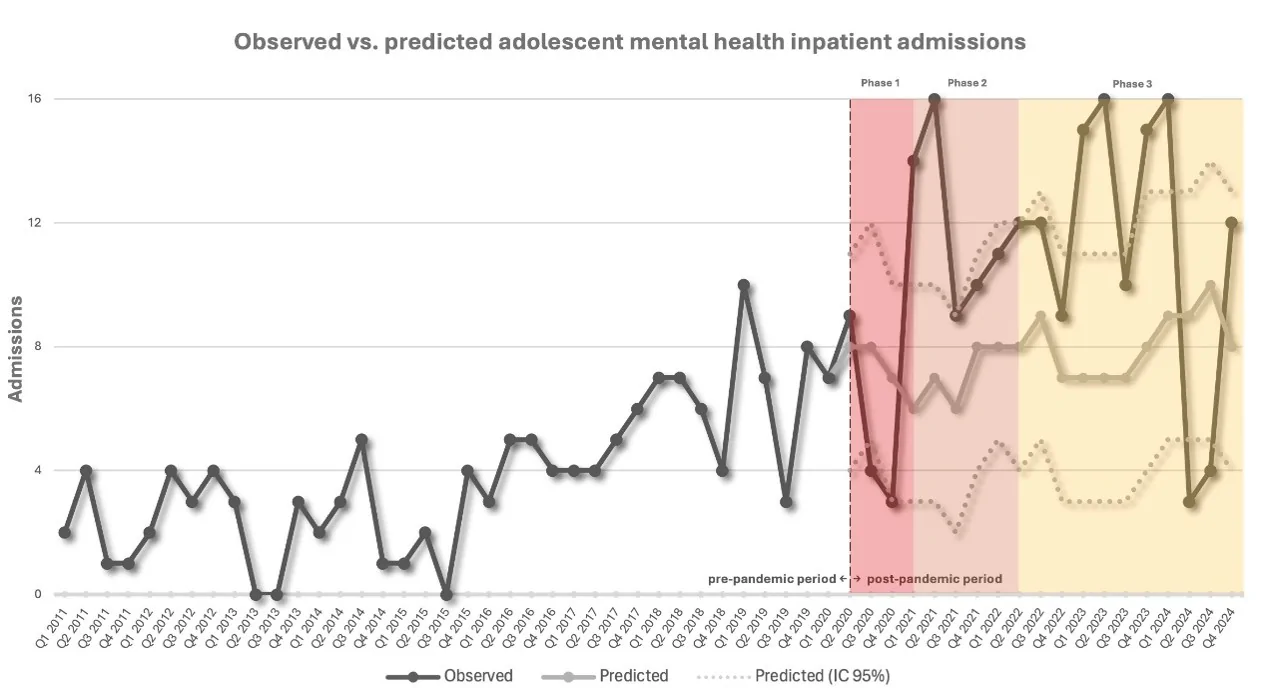
**Observed post-pandemic adolescent IMHU admissions versus SARIMA counterfactual projections**. IMHU, Inpatient Mental Health Unit; SARIMA, seasonal autoregressive integrated moving average.

### Differences in the Clinical Profile of Children and Adolescents Admitted to IMHU Before and After the Onset of the Pandemic

Of the 338 admissions to the IMHU between 2011 and 2024, 230 were from different patients, with 108 of these episodes (32.0%) constituting re-admissions. The cohort used for clinical characterisation was therefore restricted to these 230 unique patients, in order to avoid over-representation of individuals with multiple IMHU admissions. The median age of the sample was 16 years (IQR = 14–17), and it was predominantly composed of females (n = 141; 61.3%). The most common sociodemographic profile was: European/Caucasian ethnicity (n = 199; 86.5%); living with a single parent (n = 107; 46.5%); being enrolled in school or engaged in some form of vocational activity (n = 181; 78.7%); and not being in a romantic relationship (n = 202; 87.8%). A history of trauma was documented in one-third of patients (n = 77; 33.5%), 35.7% (n = 82) had a first-degree family history of mental disorder, and 23.9% (n = 55) had a relevant medical or surgical history. Substance abuse was recorded in 28.3% (n = 65). Most patients had a prior history of mental health follow-up before admission to the IMHU (n = 194; 84.3%). The most frequent reason for admission was suicidal behaviour (n = 130; 56.5%), the most common treatment modality was combined psychotherapy and pharmacotherapy (n = 93; 40.4%), and the median length of stay was three days (IQR = 1–6). Anxiety disorders were the most frequent primary ICD-10 diagnosis at discharge (n = 42; 18.3%), and most patients were referred to the child and adolescent mental health team after discharge (n = 185; 80.4%). Further details on the sociodemographic and clinical profile of the children and adolescents, as well as differences between the pre- and post-pandemic periods, can be found in Table 1.

**Table 1.** **Clinical characterisation of adolescent IMHU admissions, and differences between the pre- and post-pandemic periods**.

		Total (n = 230)	Pre-pandemic period (n = 95)	Post-pandemic period (n = 135)	*p*-value
Age (years), median (IQR)		16 (IQR 14–17)	16 (IQR 15–17)	16 (IQR 14–17)	0.441 ^a^
Gender (female), n (%)		141 (61.3%)	47 (49.5%)	94 (69.6%)	0.002 ^b^
Ethnicity (European Caucasian), n (%)		199 (86.5 %)	83 (87.4%)	116 (85.9%)	0.846 ^b^
Living arrangement (with one parent), n (%)		107 (46.5%)	37 (38.9%)	70 (51.9%)	0.154 ^b^
Occupational status (active/studying), n (%)		181 (78.7%)	74 (77.9%)	107 (79.3%)	0.870 ^b^
Relationship status (single), n (%)		202 (87.8%)	81 (85.3%)	121 (89.6%)	0.413 ^b^
First-degree family history of mental disorder (yes), n (%)		82 (35.7%)	35 (36.8%)	47 (34.8%)	0.781 ^b^
History of traumatic experiences (yes), n (%)		77 (33.5%)	30 (31.6%)	47 (34.8%)	0.671 ^b^
History of substance use (yes), n (%)		65 (28.3%)	29 (30.5%)	36 (26.7%)	0.554 ^b^
Relevant somatic or surgical history (yes), n (%)		55 (23.9%)	20 (21.1%)	35 (25.9%)	0.435 ^b^
Prior follow-up in mental health services (yes), n (%)		194 (84.3%)	82 (86.3%)	112 (83.0%)	0.582 ^b^
Primary reason for admission, n (%)					<0.001 ^b^
	Suicidal behaviour	130 (56.5%)	38 (40.0%)	92 (68.1%)	
	Behavioural disturbances	56 (24.3%)	33 (34.7%)	23 (17.1%)	
	Other clinical reasons	44 (19.1%)	24 (25.3%)	20 (14.8%)	
Type of treatment received during admission, n (%)					<0.001 ^b^
	Combined psychopharmacological and psychotherapeutic treatment	93 (40.4%)	58 (61.1%)	35 (25.9%)	
	Psychopharmacological treatment	91 (39.6%)	22 (23.2%)	69 (51.1%)	
	Psychotherapeutic treatment	46 (20.0%)	15 (15.7%)	31 (23.0%)	
Length of stay (days), median (IQR)		3 (IQR 1–6)	4 (IQR 2–7)	2 (IQR 1–5)	<0.001 ^a^
Main diagnosis at discharge (ICD-10 codes), n (%)					0.084 ^c^
	Substance use disorders (F10-19)	13 (5.7%)	9 (9.5%)	4 (3%)	
	Schizophrenia and related psychotic disorders (F20-29)	31 (13.5%)	15 (15.8%)	16 (11.9%)	
	Mood disorders (F30-39)	37 (16.1%)	11 (11.6%)	26 (19.3%)	
	Anxiety disorders (F40-48 plus F93)	42 (18.3%)	12 (12.6%)	30 (22.2%)	
	Eating disorders (F50)	14 (6.1%)	4 (4.2%)	10 (7.4%)	
	Personality disorders (F60-69)	28 (12.2%)	12 (12.6%)	16 (11.9%)	
	Intellectual disability (F70-79) and Autism spectrum disorders (F84.x)	31 (13.5%)	13 (13.7%)	18 (13.3%)	
	Behavioral and emotional disorders (F90-98 excluding F93)	28 (12.2%)	16 (16.8%)	12 (8.9%)	
	Other and unspecified ICD-10 mental disorders	6 (2.6%)	3 (3.2%)	3 (2.2%)	
Type of follow-up after discharge (Child and adolescent mental health unit), n (%)		185 (80.4%)	80 (84.2%)	105 (77.8%)	0.435 ^b^

Abbreviations: ICD-10, International Classification of Diseases 10th Revision; IQR, interquartile range; IMHU, Inpatient Mental Health Unit. ^a^ Mann–Whitney U test. ^b^ Chi-square test. ^c^ Fisher–Freeman–Halton exact test.

More than half of the adolescents (n = 135; 58.7%) were admitted to the IMHU during the period following the onset of the pandemic (Q2 2020 to Q4 2024), compared to 41.3% (n = 95) who were admitted during the pre-pandemic period (Q1 2011 to Q1 2020). Bivariate analysis revealed statistically significant differences between the pre- and post-pandemic periods with regard to gender, primary reason for admission, type of treatment received and length of hospitalisation, whereas the distribution of ICD-10 diagnoses at discharge showed marginal differences. Thus, the post-pandemic period was characterised by a higher proportion of female admissions, increasing from 49.5% (n = 47) before the pandemic, to 69.6% (n = 94) afterwards (*p* = 0.002). Suicidal behaviour also became substantially more frequent as the primary reason for admission, rising from 40.0% (n = 38) in the pre-pandemic period, to 68.1% (n = 92) in the post-pandemic period (*p* < 0.001). Type of treatment received during admission also changed significantly (*p* < 0.001), with exclusively psychopharmacological treatment becoming the most frequent approach after the pandemic onset (n = 69; 51.1%), while the use of combined psychotherapy and pharmacotherapy decreased from 61.1% (n = 58) to 25.9% (n = 35). Finally, length of stay decreased significantly after the onset of the pandemic, from a median of 4 days (IQR = 2–7) to 2 days (IQR = 1–5; *p* < 0.001). The overall distribution of ICD-10 diagnoses at IMHU discharge showed only marginal differences between periods (*p* = 0.084). Descriptive and exploratory observations of individual diagnostic categories revealed an increase in mood disorders, rising from 11.6% (n = 11) of admissions prior to the pandemic to 19.3% (n = 26), as well as an increase in anxiety disorders, rising from 12.6% (n = 12) to 22.2% (n = 30). There was also a substantial rise in eating disorders, which increased from 4.2% (n = 4) to 7.4% (n = 10). Conversely, a decline was seen in substance use disorders, falling from 9.5% (n = 9) to 3.0% (n = 4), and in behavioural and emotional disorders, dropping from 16.8% (n = 16) to 8.9% (n = 12). The proportions of psychotic disorders, personality disorders, autism spectrum disorders/intellectual disability and other unspecified mental disorders remained relatively stable across both periods, with only minor percentage variations.

The aforementioned significant and trend-level variables were entered into a logistic regression model to examine whether they were independently associated with the post-pandemic period. Age, living arrangement and prior history of mental health follow-up were also included as covariates, since these factors may have been impacted by the pandemic due to potential changes in family structure and disruptions to the early detection and intervention of mental health issues. Length of stay was excluded to avoid overadjustment, since it can be considered a downstream feature of the clinical episode of admission. Prior to fitting the model, several variables were recoded to improve parsimony and clinical interpretability: the primary reason for admission was dichotomised into “suicidal behaviour” versus “other reasons”; living arrangement was recoded as “living with both parents” versus “living with one parent or other arrangements”; the variable “treatment received during admission” was dichotomised as “only psychopharmacological treatment” versus “other treatments”; and ICD-10 diagnoses were recoded as “internalising disorders” (including mood disorders, anxiety disorders, and eating disorders) versus “other diagnoses”. This latter grouping was based on prior clinical and epidemiological knowledge, as discussed in the Introduction, regarding the growing prominence of internalising symptoms in this age group. Multivariable logistic regression analysis revealed that the overall model was statistically significant (χ^2^ = 50.003, df = 7, *p* < 0.001), and the Hosmer–Lemeshow test indicated that it fitted the data adequately (χ^2^ = 12.133, df = 8, *p* = 0.145). No relevant collinearity was identified between the included covariates. After adjustment, admission during the post-pandemic period, compared with the pre-pandemic period, was independently associated with suicidal behaviour as the primary reason for admission (OR = 2.53, 95% CI 1.33–4.83, *p* = 0.005), having an internalising disorder (OR = 1.98, 95% CI 1.03–3.79, *p* = 0.040), and receiving only psychopharmacological treatment (OR = 4.61, 95% CI 2.42–8.80, *p* < 0.001). No independent associations were found for age, gender, living arrangements or prior specialised mental health follow-up. A summary of the regression analysis is shown in Table 2.

**Table 2.** **Multivariable logistic regression analysis of IMHU admission-related factors independently associated with the post-pandemic period**.

Variable	OR	95% CI	*p*-value
Age	0.92	0.74–1.15	0.461
Female gender	1.55	0.80–3.03	0.195
Living with one parent or other arrangements	1.47	0.79–2.75	0.229
Prior specialised mental health follow-up	0.58	0.25–1.35	0.207
Suicidal behaviour as primary reason for admission	2.53	1.33–4.83	0.005
Only psychopharmacological treatment during admission	4.61	2.42–8.80	<0.001
Internalising disorders*	1.98	1.03–3.79	0.040

Abbreviations: OR, odds ratio; CI, confidence interval; IMHU, Inpatient Mental Health Unit. *Internalising disorders: mood disorders, anxiety disorders, and eating disorders. Model summary: χ^2^ = 50.003, df = 7, *p* < 0.001; Hosmer–Lemeshow χ^2^ = 12.133, df = 8, *p* = 0.145.

## Discussion

The present study aimed to examine long-term trends and changes in the clinical profile of adolescent admissions to IMHU, and to explore the temporal impact of the COVID-19 pandemic on these admissions. To this end, a time-series modelling analysis was conducted, covering the period from 2011 to 2024. The sociodemographic and clinical profiles of adolescents admitted before and after the pandemic were then compared. The time-series analysis revealed that, although an upward trend in mental health admissions had already been observed prior to the pandemic, its onset was associated with a further statistically significant increase in hospitalisations. This equated to an average of 2.71 additional admissions per quarter exceeding what would have been expected based on historical trends prior to the pandemic. Observed admissions exceeded the model’s point estimate in 79% of post-pandemic quarters, whereas 31.5% of quarters exceeded the upper limit of the 95% forecast CI. Overall, the post-pandemic period showed a relative increase of 36.1% in adolescent IMHU admissions. Moreover, the proportion of readmission episodes remained similar in both timeframes, suggesting that the post-pandemic surge in IMHU admissions was due to a general increase, rather than being an artefact of repeated admissions. Alongside this increase, the findings revealed a shift in the clinical characterisation of adolescents admitted to IMHU. Significant differences were observed between the pre- and post-pandemic periods in terms of gender (a higher proportion of females after the pandemic), primary reasons for admission (an increase in hospitalisations due to suicidal behaviour), treatment received (greater reliance on predominantly psychopharmacological approaches) and length of stay (shorter after the pandemic), whereas trend-level differences were observed in ICD-10 diagnoses at discharge (numerical increases in mood, anxiety, and eating disorders, and decreases in substance use, behavioural, and emotional disorders). However, of these variables, suicidal behaviour as the primary reason for admission, having an internalising disorder, and receiving exclusively psychopharmacological treatment during hospitalisation were the only clinical-related variables independently associated with admission during the post-pandemic period, compared with the pre-pandemic period, in the multivariable analysis. This study adds to the limited body of evidence using time-series analysis to examine changes in adolescent mental health hospitalisation trends before and after the pandemic, alongside a detailed comparison of clinical profiles between both periods.

Overall, the results of this study were consistent with previous reports in the international literature. The significant increase in adolescent admissions to the IMHU in the post-pandemic period, compared to the counterfactual projection based on pre-existing historical trends, was aligned with the majority of published studies on this topic. These studies have described a significant rise in paediatric mental health hospitalisations since 2021, following an initial decrease linked to lockdown measures [12, 35, 36]. However, our exploratory sub-period analysis suggested that the excess in IMHU admissions observed after the onset of the pandemic did not follow a homogeneous pattern over time and appeared to attenuate in 2024, when observed admissions were in line with counterfactual expectations. In terms of sociodemographic and clinical profiles, the increased proportion of female adolescents admitted to IMHU in the post-pandemic period was also consistent with the findings of other studies, which have documented a higher proportion of females among IMHU hospitalisations and visits to the mental health emergency department [15, 37, 38, 39]. Nevertheless, the loss of statistical significance for female gender in our adjusted multivariable model suggested that this gender difference may have been partly accounted for by other admission-related clinical features that became more prominent after the onset of the pandemic. In the same vein, the increase in hospital admissions due to suicidal behaviour observed in this study aligned with the widely reported global trend of rising self-harm and suicide attempts among children and adolescents since the onset of the COVID-19 [35, 36, 40, 41, 42]. By contrast, the reduction in mean length of stay observed in our post-pandemic cohort should be framed within a mixed and heterogeneous body of evidence. Thus, studies examining paediatric psychiatric length of stay after the pandemic have reported inconsistent findings, including decreases, relative stability, and, in some cases, longer hospitalisations [12, 38, 43, 44]. The increase in pharmacological interventions observed in our sample was consistent with the general increase in the prescription of psychopharmacological treatments for children and adolescents following the onset of the pandemic, particularly anxiolytics and antidepressants [44, 45, 46, 47]. Finally, with regard to the differences in ICD-10 diagnoses at discharge between the pre- and post-pandemic periods, the increase in internalising diagnoses (i.e., mood disorders, anxiety disorders, and eating disorders) observed in our study was consistent with previous research [15, 37, 48, 49]. However, the decrease in externalising and substance use disorders, alongside the stability of psychotic, personality, autism spectrum, and intellectual disability disorders, was only partly consistent with previous reports, which have described heterogeneous diagnostic patterns among IMHU admissions [38, 49, 50, 51].

### Methodological Limitations

Several limitations should be considered when interpreting the results of this study. Firstly, although the time-series forecasting design allowed us to identify a statistically significant deviation from historical trends, direct causality cannot be inferred from these data. Therefore, the observed post-pandemic surge in adolescent IMHU admissions should be interpreted with caution, as unmeasured contextual factors such as changes in referral pathways, diagnostic practices, service organisation, bed availability or local mental health policies may have contributed to our findings. This must be considered a limitation in interpreting the data. In addition, the magnitude of this estimated excess in admissions should be considered in light of the model’s forecasting uncertainty, especially given its relatively high MAPE. Furthermore, while the findings largely align with those of previous research, the observational, single-centre nature of this study limits its generalisability to other healthcare systems, particularly those operating outside the Spanish public health framework. The study was also retrospective and reliant on EHRs, with information on the study variables derived exclusively from clinical notes. Depending on the completeness of the EHRs and the methods of data recording by healthcare professionals involved in the adolescents’ care, there is a possibility that this may have resulted in an underestimation or overrepresentation of certain phenomena [52]. A further limitation was the absence of standardised psychometric data with which to assess symptom severity or estimate the adolescents’ premorbid functioning. Although “proxy” instruments exist that would allow their retrospective psychometric assessment based on information contained in the EHRs, these tools were not utilised in the present study [53, 54, 55]. As the study focused specifically on hospitalisations of adolescents in the IMHU, it was not possible to include other paediatric patients admitted to the hospital for mental health reasons outside of the IMHU (e.g., admissions to the endocrinology ward for eating disorders, or to the traumatology, intensive care, or internal medicine wards for complications resulting from suicide attempts). These cases were managed by the child and adolescent mental health liaison team and were therefore outside the scope of our IMHU sampling frame. Since systematic admission data for these cases was unavailable throughout the study period, it was not possible to reliably estimate the magnitude of these exclusions, nor determine whether they differed between the pre- and post-pandemic periods. This must be considered another limitation, which may have led to a possible underestimation of non-IMHU mental health hospitalisations and the true burden of certain diagnoses and reasons for admission. Finally, it should be noted that ICD-10 clinical diagnoses were carried out within the framework of routine clinical practice, without confirmation through structured diagnostic interviews and without subsequent longitudinal validation or review, which should be considered an additional limitation.

### Implications and Future Directions

Despite the previously mentioned limitations, this study has important implications. The catalytic effect of the COVID-19 pandemic on the mental health of children and adolescents highlights their distinctive vulnerability to adverse circumstances. The increase in hospital admissions due to suicidal behaviour, depressive disorders, anxiety disorders, and eating disorders indicates a clear rise in demand for mental healthcare services. Although our observational data cannot substantiate the underlying psychosocial mechanisms directly, this clinical trend is consistent with the hypothesis that effective coping mechanisms are weakening within this age group [56]. Consequently, our findings prompt reflection on the apparent diminished resilience in adolescents, a phenomenon that had already been noted from a sociological perspective prior to the pandemic [57]. They also further reinforce the need to systematically develop and implement interventions aimed at strengthening coping skills and providing psychoeducation for families. Such strategies are essential for enhancing young people’s capacity to respond to an increasingly fragmented social fabric and a context characterised by greater digital exposure [29, 58]. Moreover, the increase in paediatric mental health admissions, together with the shift in clinical presentation towards a predominance of internalising symptoms, highlights the need to rethink the organisation of IMHU and its role within the broader network of child and adolescent mental health services. In this context, key strategies to ensure safe, developmentally appropriate therapeutic environments should include establishing specialised adolescent mental health inpatient wards (either as standalone units or clearly differentiated, age-appropriate areas) and strengthening community-based services and stepped-care pathways [59]. Nonetheless, the applicability of these recommendations may vary according to local healthcare organisation, resource availability, referral pathways, inpatient service capacity, and sociocultural context. Finally, the increased use of pharmacological treatments among children and young people admitted to IMHU after the pandemic suggests a possible shift in therapeutic interventions towards more medicalised strategies. However, it is important to interpret this observed increase with caution, as the choice of treatment modality was decided during admission, and therefore represented a downstream clinical feature rather than an antecedent predictor of post-pandemic hospitalization. This association was likely to have been influenced by residual confounding factors related to illness severity, diagnostic complexity, and clinical urgency. In particular, the higher proportion of admissions involving internalising disorders with suicidal behaviour may have contributed to the greater use of psychopharmacological treatment for rapid symptom control and short-term risk management during hospitalisation, while psychotherapeutic interventions would be expected to be initiated after discharge in outpatient child and adolescent mental health services. Nevertheless, this trend highlights the need to consider the risk of placing too much emphasis on psychotropic medications in IMHU, at the expense of psychological and psychosocial interventions [60]. In this regard, it is essential to ensure that the clinical management of adolescent distress maintains an appropriate balance between pharmacological treatment, individual and family-based psychotherapeutic approaches, school and community-based interventions, and preventive strategies, thereby avoiding hospitalisation and medication becoming the dominant clinical response to mental health problems in young people. Future research should assess whether the upward trend in hospitalisations for mental health reasons among adolescents will continue in the coming years, and whether the predominant clinical profile identified in this study will persist over time. To address these questions, studies with longer time series are required to determine whether the observed trends reflect a transient phenomenon linked to the pandemic period, or a structural shift in demand for mental health services among children and adolescents. Furthermore, longitudinal studies are essential for examining the medium- and long-term outcomes of adolescents admitted to IMHU during the pandemic, including symptomatic and functional recovery, chronicity, and associated medical morbidity.

## Conclusions

The COVID-19 pandemic not only overlapped with an already increasing trend in mental health hospitalisations among adolescents, but was also associated with an additional and statistically significant rise in admissions, alongside a shift in the clinical profile towards a clear predominance of female adolescents presenting with internalising disorders and suicidal behaviour. This pattern, together with shorter lengths of stay and a greater reliance on pharmacological interventions, highlights the particular vulnerability of children and adolescents in contexts of adversity. It also emphasises the importance of strengthening and adapting inpatient and community mental health services for adolescents, and of promoting integrated care models that combine psychopharmacological, psychotherapeutic, and psychosocial interventions.

## Availability of Data and Materials

The data that support the findings of this study are available on reasonable request from the corresponding author. The data are not publicly available due to privacy or ethical restrictions.
